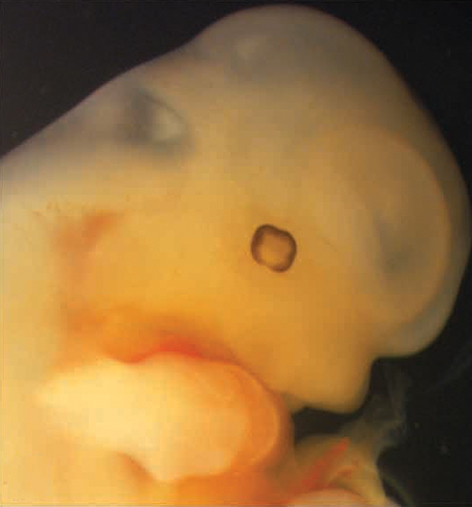# Effects of maternal diabetes on the Wnt-PCP pathway and embryogenesis

**Published:** 2015-02

Congenital malformations in diabetic pregnancy are still a major health concern despite the improvements in glycaemic control strategies. Advancing our understanding of the mechanisms responsible for diabetic embryopathies could help develop strategies to prevent this condition. It is known that maternal hyperglycaemia affects components of the Wnt-PCP signalling pathway, including the dishevelled-associated activator of morphogenesis 1 gene (*Daam1*), which is important for the morphogenesis of the eye and other organs. In this study, Patricia Ybot-González’s group sought to further explore the role of the Wnt-PCP pathway in diabetic embryopathies. They induced experimental diabetes in pregnant mice and found that maternal hyperglycaemia induced eye malformations in mouse embryos. *In vitro* studies confirmed sensibility of eye development to hyperglycaemia and showed reduced expression of Wnt-PCP pathway genes. Notably, *Daam1*-mutant embryos displayed similar eye malformations, which were worsened by environmental hyperglycaemia. These results show that maternal diabetes can affect embryogenesis by altering Wnt-PCP signalling. Modulating this pathway might thus represent a potential strategy to reduce the impact of diabetic embryopathies. **Page 157**

**Figure:**